# Supplementary material for: Rational design of aptamer switches with programmable pH response
Source: Nat Commun. 2020 Jun 10;11:2946. doi: 10.1038/s41467-020-16808-2 (PMC7286914; doi:10.1038/s41467-020-16808-2)
Supplement: Supplementary file 1 — Supplementary Information [file 41467_2020_16808_MOESM1_ESM.pdf]

Supplementary Information

Rational Design of Aptamer Switches with Tunable pH Response

Ian A.P. Thompson et al.

Contents

Supplementary Figure 1 | Three-state population shift model of aptamer binding and predicted effects of construct design on binding curves
Supplementary Figure 2 | pH Titrations of triplex sequences in the absence of ligand
Supplementary Figure 3 | Three-state binding model for triplex constructs
Supplementary Figure 4 | Normalized pH-dependent binding curves for all triplex constructs

Supplementary Figure 5 | Three-state binding model for DS mismatch constructs
Supplementary Figure 6 | Normalized pH-dependent binding curves for A-G mismatch construct
Supplementary Figure 7 | Normalized pH-dependent binding curves for a construct combining triplex and mismatch control elements
Supplementary Figure 8 | Secondary structure optimization results for triplex sequences

Supplementary Table 1 | Sequences of all constructs used in this work

Supplementary Methods

Supplementary References

Supplementary Figures

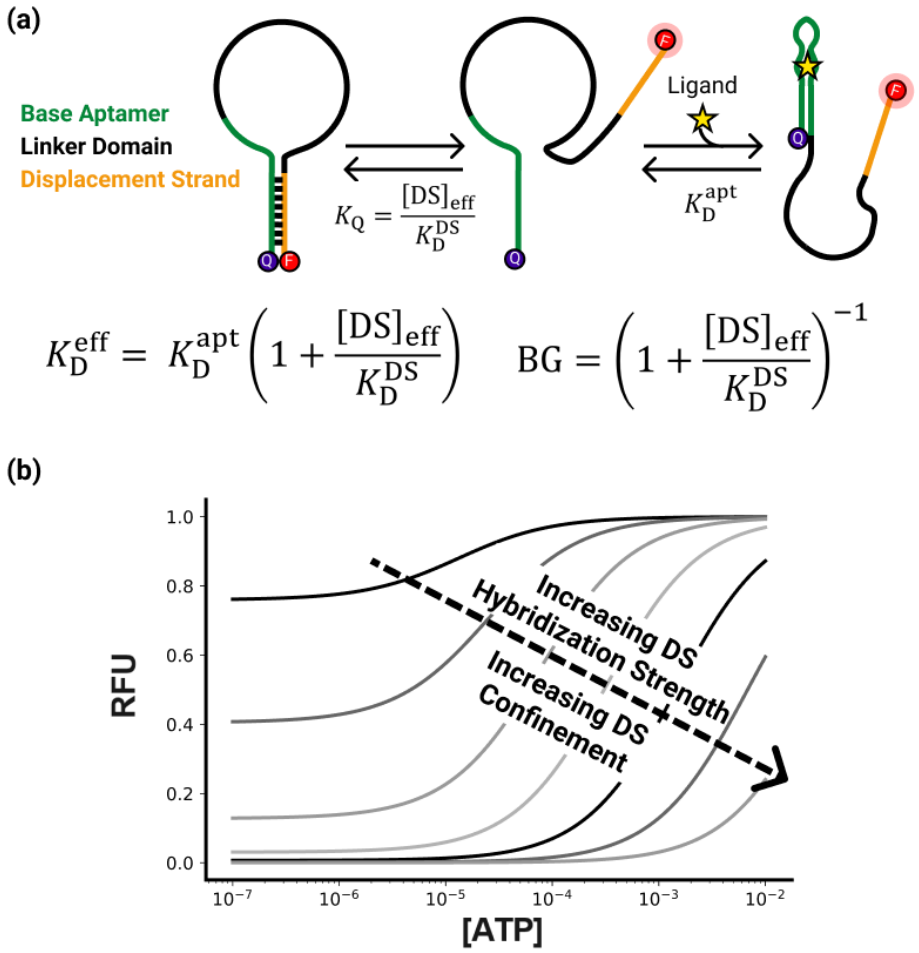

Supplementary Figure 1 | Three-state population-shift aptamer binding model and predicted effects of construct design on binding curves. **(a)** The three-state population shift model underlies the PSD construct mechanism, where hybridization of the displacement strand (DS) with equilibrium constant K_Q inhibits aptamer binding. Target binding shifts the equilibrium away from the DS hybridized state, leading to increased fluorescence and allowing for optical quantification of target binding. The construct's effective binding affinity K_D^{eff} and the magnitude of background signal both depend on the DS effective concentration, $[DS]_{eff}$, and hybridization strength, K_D^{DS}.[1] **(b)** Effects of varying the DS effective concentration or hybridization strength on the predicted binding curves from the three-state model.

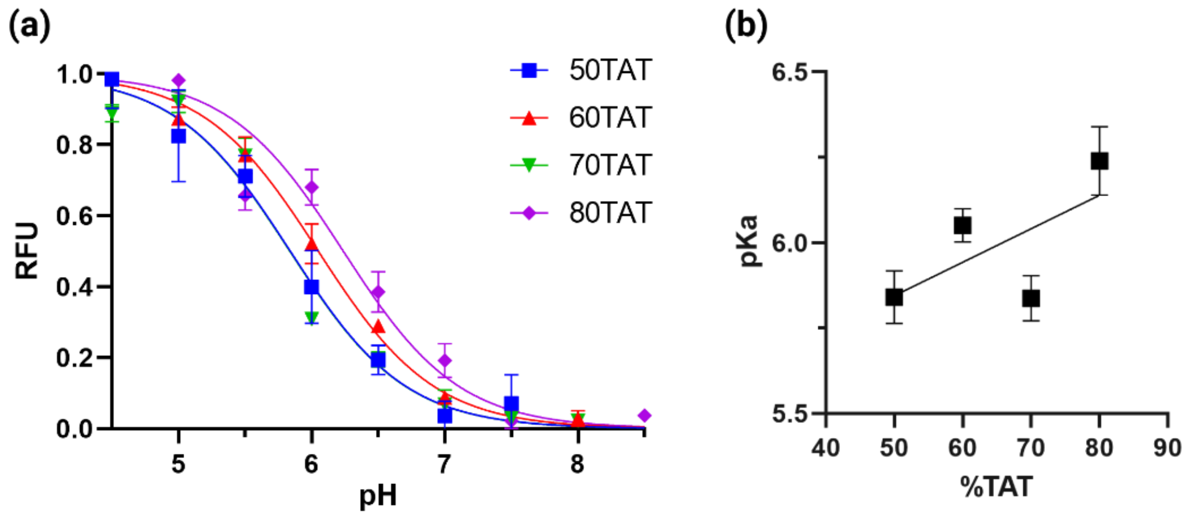

Supplementary Figure 2 | pH Titrations of triplex sequences in the absence of ligand. **(a)** Folding-induced fluorescence of constructs with varying TAT triplet compositions at range of pH values for [ATP] = 0 shows. **(b)** Increasing the TAT content of the triplex motif predictably shifts the pK_a of the switch toward more neutral pH. Analysis of data in **(a)** is described in **Supplementary Methods.** Data points and error bars in **(a)** show the means and standard deviations of n=3 independent experiments. Data points and error bars in **(b)** show the best fit values and standard deviations extracted from fits to binding curve data from n=3 independent experiments using non-linear least squares fitting. Source data are provided as a Source Data file.

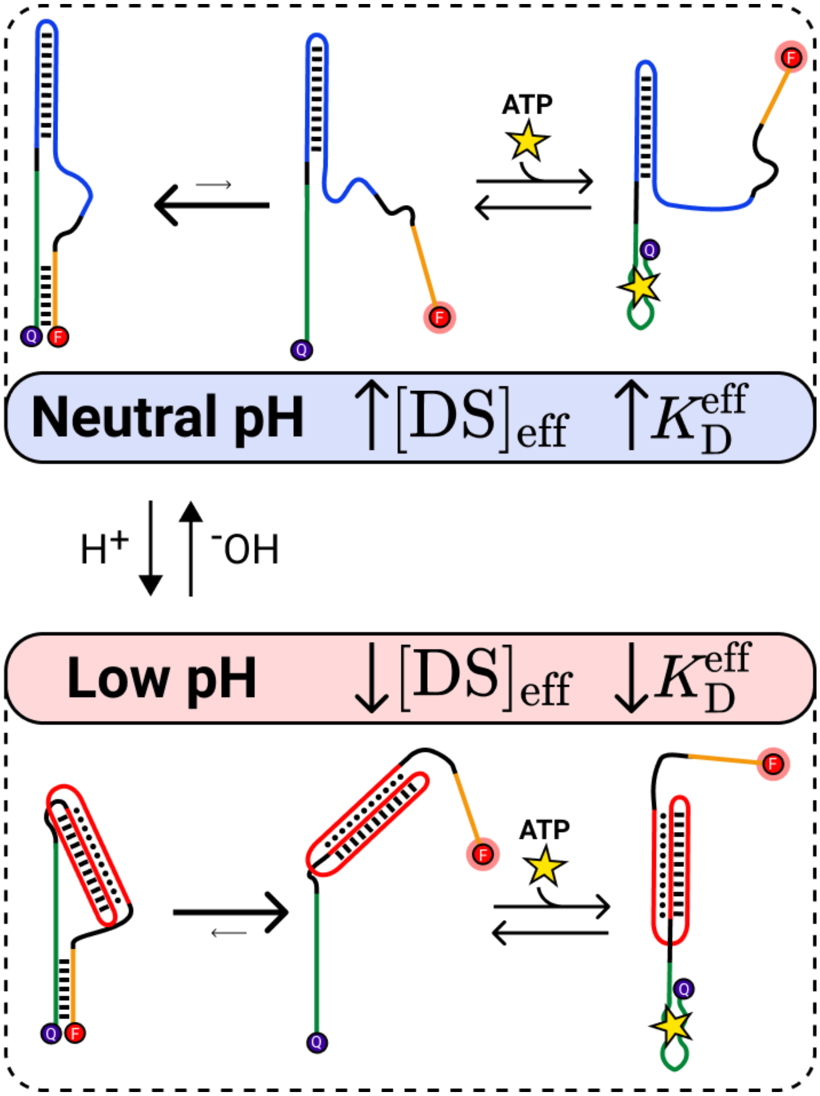

Supplementary Figure 3 | Three-state binding model for triplex constructs. The PSD construct mechanism relies upon pH-dependent shifts in the DS hybridization equilibrium to shift the construct's overall $K_{\mathrm{D}}^{\mathrm{eff}}$. Selective binding at low pH is achieved through pH-dependent conformational changes in the triplex motif, which lead to changes in $[\mathrm{DS}]_{\mathrm{eff}}$—inhibiting DS hybridization at low pH, and enhancing it at neutral pH. Equilibrium is shifted toward the hybridized state at neutral pH (top) and toward the unbound state at low pH (bottom), which selectively increases target affinity under the latter condition.

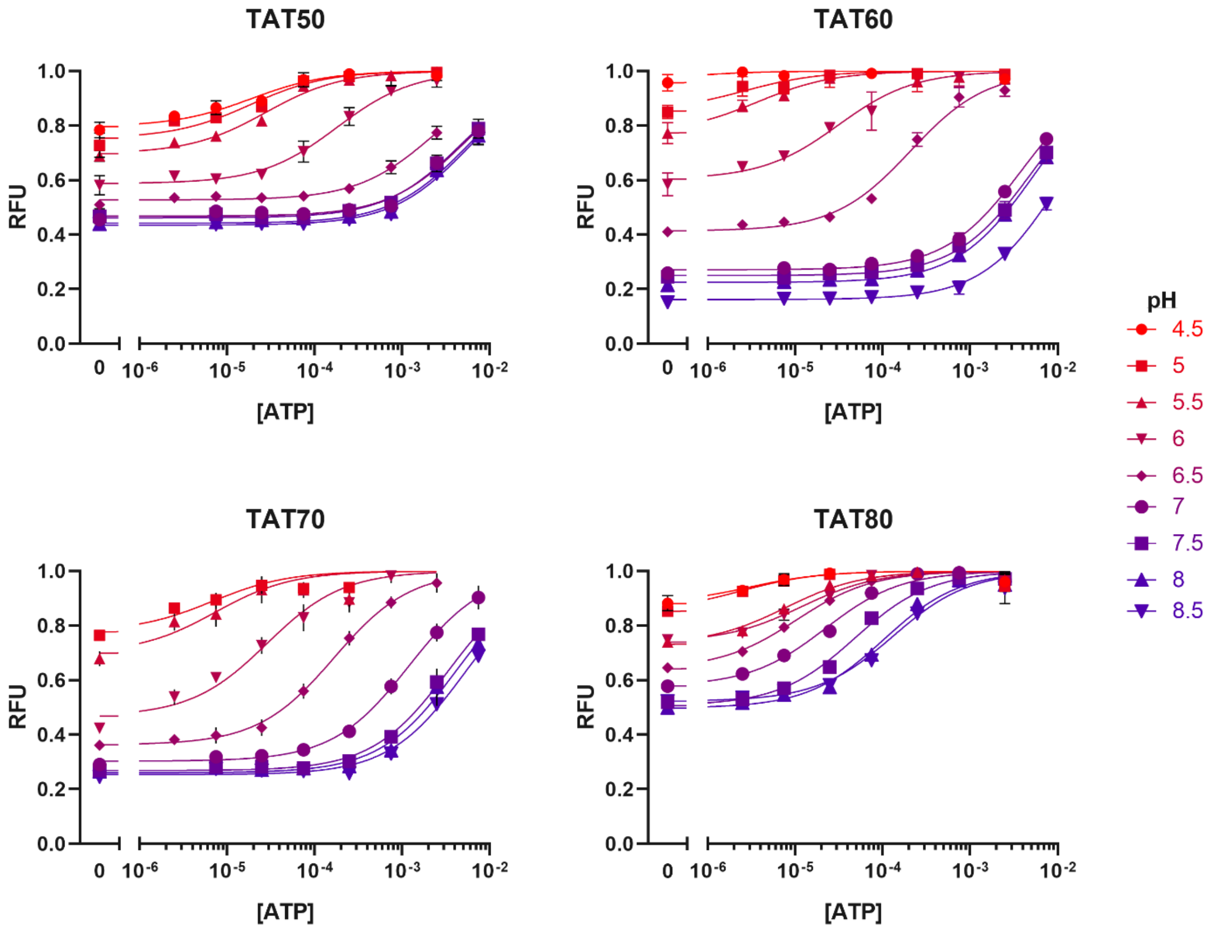

Supplementary Figure 4 | Normalized pH-dependent binding curves for all triplex constructs. Plots show normalized PSD construct fluorescence vs. ATP concentration at a range of solution pH values. The normalization procedure is given in Methods. Data points and error bars in show the means and standard deviations of n=3 independent experiments. Source data are provided as a Source Data file.

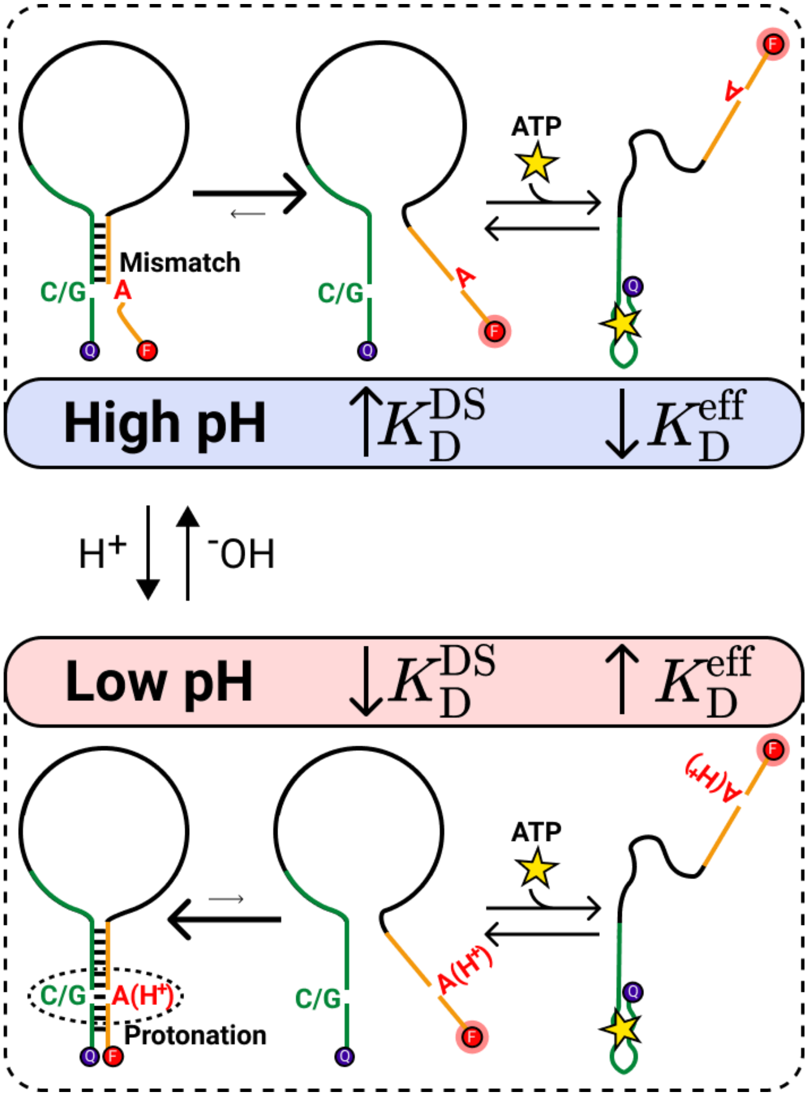

Supplementary Figure 5 | Three-state binding model for DS mismatch constructs. High-pH-selective binding is achieved through pH-dependent changes in DS hybridization strength due to the formation of protonated A-C or A-G mismatches. At high pH (top), the inserted mismatch remains unprotonated and does not bond, leading to weak DS hybridization (high K_D^{DS}) and shifting equilibrium towards the unbound state. Upon protonation at acidic pH (bottom), DS hybridization is strengthened (decreased K_D^{DS}) by formation of either an $A(H^+)$-C wobble base pair or an $A(H^+)$-G Hoogsteen base pair, which shifts equilibrium towards the hybridized state.

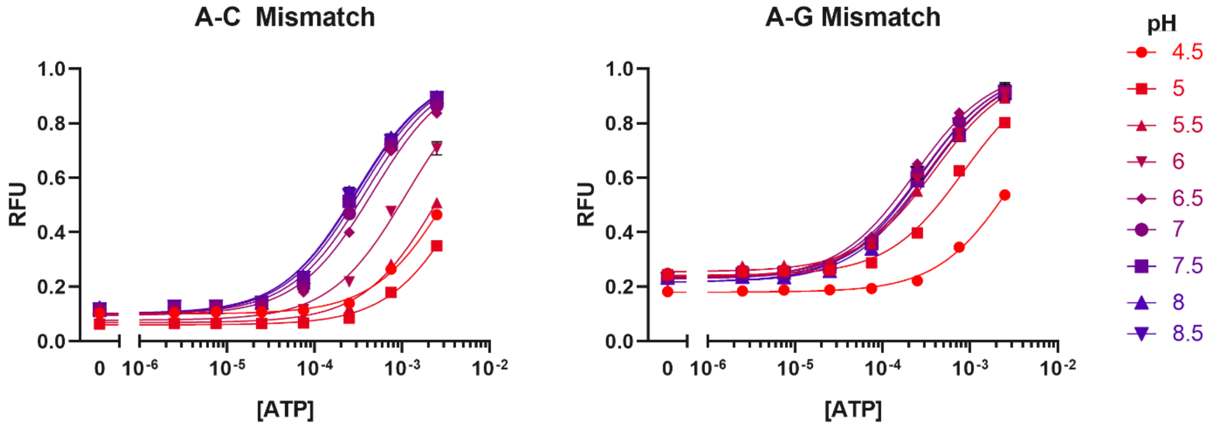

Supplementary Figure 6 | Normalized pH-dependent binding curves for all DS mismatch constructs. Plots show normalized PSD construct fluorescence vs. ATP concentration at a range of pH values. The normalization procedure is given in Methods. Data points and error bars in show the means and standard deviations of n=3 independent experiments. Source data are provided as a Source Data file.

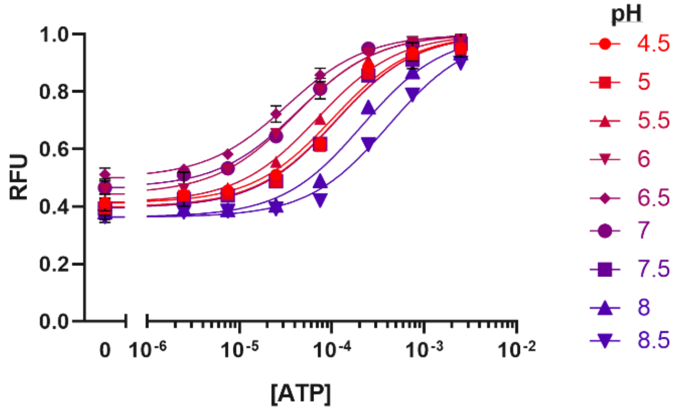

Supplementary Figure 7 | Normalized pH-dependent binding curves for a construct combining triplex and mismatch control elements. The tested construct incorporates both the TAT80 linker and an A-C mismatch within the displacement strand. The plot shows normalized construct fluorescence vs. ATP concentration at a range of pH values. The normalization procedure is given in Methods. Data points and error bars in show the means and standard deviations of n=3 independent experiments. Source data are provided as a Source Data file.

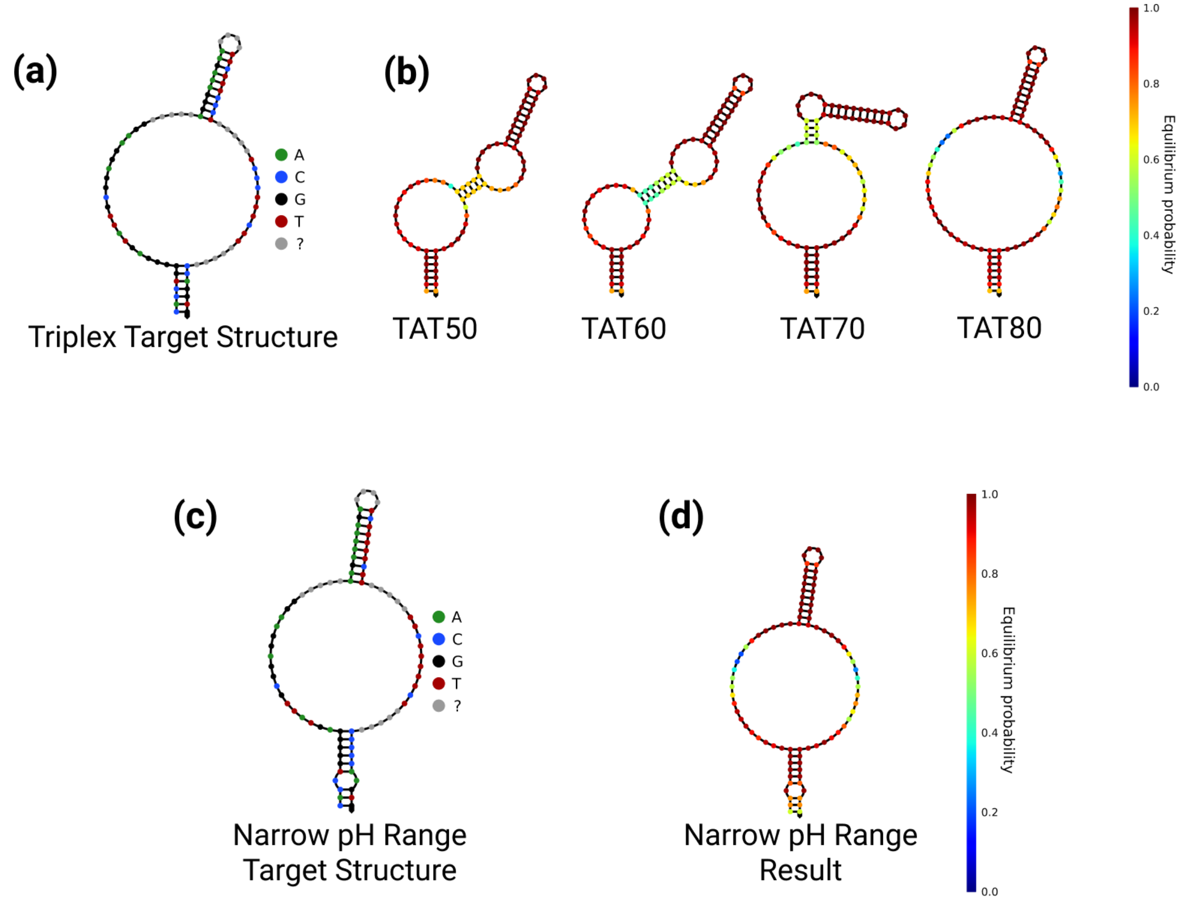

Supplementary Figure 8 | Secondary structure optimization results for triplex sequences. **(a)** The target secondary structure for our triplex-based PSD design was set to achieve DS hybridization to the aptamer and to allow for duplex formation between two strands of the intramolecular triplex motif. Random regions (gray bases) were optimized to minimize unintended interactions between the linker motif and aptamer. **(b)** Resulting structures of the four triplex-based PSD constructs obtained through optimization closely match the target structure, with minimized interference between the triplex motif and aptamer. **(c)** Target secondary structure and **(d)** resulting optimized secondary structure for the narrow pH range construct. All optimized structures shown were obtained through simulation in the NUPACK web application using conditions $[Na^+]$ = 50 mM and $[Mg^{2+}]$ = 6 mM at 25 °C.[2]

Construct		Sequence
ATP PSD with no triplex motif		/5IABkFQ/CACCTGGGGGAGTATTGCGGAGGAAGGTTTTTTTTTTTTTTTTCCA GGTG/3Cy3Sp/
TAT50 Triplex	Constraint	CACCTGGGGGAGTATTGCGGAGGAAGG *NNNNN* AGGGGAAGAA *NNNN* TTCTTCCCCT *NNNNN* TCCCCTTCTT *NNNNN* CCAGGTG
	Optimized	/5IABkFQ/CACCTGGGGGAGTATTGCGGAGGAAGG TTTTC AGGGGAAGAA GCAC TTCTTCCCCT TTTTT TCCCCTTCTT TTAAA CCAGGTG/3Cy3Sp/
TAT60 Triplex	Constraint	CACCTGGGGGAGTATTGCGGAGGAAGG *NNNNN* AGGGAAAGAA *NNNN* TTCTTTCCCT *NNNNN* TCCCTTTCTT *NNNNN* CCAGGTG
	Optimized	/5IABkFQ/CACCTGGGGGAGTATTGCGGAGGAAGG TTTTG AGGGAAAGAA TCAT TTCTTTCCCT ATGTT TCCCTTTCTT TTTAA CCAGGTG/3Cy3Sp/
TAT70 Triplex	Constraint	CACCTGGGGGAGTATTGCGGAGGAAGG *NNNNN* AGAAAGAAGA *NNNN* TCTTCTTTCT *NNNNN* TCTTTCTTCT *NNNNN* CCAGGTG
	Optimized	/5IABkFQ/CACCTGGGGGAGTATTGCGGAGGAAGG TTTTA AGAAAGAAGA GTGC TCTTCTTTCT CCTTT TCTTTCTTCT TTGAG CCAGGTG/3Cy3Sp/
TAT80 Triplex	Constraint	CACCTGGGGGAGTATTGCGGAGGAAGG *NNNNN* AAGAAAAAGA *NNNN* TCTTTTTCTT *NNNNN* TTCTTTTTCT *NNNNN* CCAGGTG
	Optimized	/5IABkFQ/CACCTGGGGGAGTATTGCGGAGGAAGG TTTTA AAGAAAAAGA TTGC TCTTTTTCTT ATTGT TTCTTTTTCT AGGGG CCAGGTG/3Cy3Sp/
A-C Mismatch		/5IABkFQ/CACCTGGGGGAGTATTGCGGAGGAAGG TTTTTTTTTTTTTA CCCCCCA**A**GTG/3Cy3Sp/
A-G Mismatch		/5IABkFQ/CAC CTG GGG GAG TAT TGC GGA GGA AGG TTTTTTTTTTTTTTT CCC**A**AGGTG/3Cy3Sp/
Combined TAT80/A-C Mismatch	Constraint	CACCTGGGGGAGTATTGCGGAGGAAGG *NNNNN* AAGAAAAAGA *NNNN* TCTTTTTCTT *NNNNN* TTCTTTTTCT *NNNNN* CCCCCA**A**GTG
	Optimized	/5IABkFQ/CACCTGGGGGAGTATTGCGGAGGAAGG TTTTG AAGAAAAAGA TTGC TCTTTTTCTT ATTGT TTCTTTTTCT ATTTA CCCCCA**A**GTG/3Cy3Sp/

Supplementary Table 1 | Sequences of all constructs used in this work. Bases in italics signify unconstrained bases for optimization in Nupack. Bolded and underlined bases signify DS mismatches. /5IABkFQ/ represents the 5' Iowa Black FQ quencher modification. /3Cy3Sp/ represents the 3' Cy3 fluorophore modification.

Supplementary Methods

Analysis of pH-titration data for triplex constructs

All data analysis was performed in GraphPad Prism 8.0.2. Normalized triplicate Cy3 fluorescence (F_{Cy3}) vs. pH data with [ATP] = 0M for each construct was extracted from the normalized data used in the binding curve analysis presented in Methods. Folding data (in RFU) for each construct was then normalized fit to the range 0 to 1, and was fit with a Langmuir isotherm binding model of the form

$$\mathrm{RFU} = \frac{10^{-\mathrm{pH}}}{10^{-\mathrm{pK_a}} + 10^{-\mathrm{pH}}}$$

To extract $\mathrm{pK_a}$ values.

Supplementary References

1. Wilson, B. D., Hariri, A. A., Thompson, I. A. P., Eisenstein, M. & Soh, H. T. Independent control of the thermodynamic and kinetic properties of aptamer switches. *Nat. Commun.* **10**, 5079 (2019).
2. Zadeh, J. N. *et al.* NUPACK: Analysis and design of nucleic acid systems. *J. Comput. Chem.* **32**, 170–173 (2011).
