# Supplementary material for: Rational design of aptamer switches with programmable pH response
Source: Nat Commun. 2020 Jun 10;11:2946. doi: 10.1038/s41467-020-16808-2 (PMC7286914; doi:10.1038/s41467-020-16808-2)
Supplement: Supplementary file 3 — Reporting Summary [file 41467_2020_16808_MOESM3_ESM.pdf]

nature research

Corresponding author(s): H. Tom Soh

Last updated by author(s): May 15, 2020

Reporting Summary

Nature Research wishes to improve the reproducibility of the work that we publish. This form provides structure for consistency and transparency in reporting. For further information on Nature Research policies, see our Editorial Policies and the Editorial Policy Checklist.

Statistics

For all statistical analyses, confirm that the following items are present in the figure legend, table legend, main text, or Methods section.

n/a	Confirmed	
☐	☒	The exact sample size (n) for each experimental group/condition, given as a discrete number and unit of measurement
☐	☒	A statement on whether measurements were taken from distinct samples or whether the same sample was measured repeatedly
☒	☐	The statistical test(s) used AND whether they are one- or two-sided<br>*Only common tests should be described solely by name; describe more complex techniques in the Methods section.*
☒	☐	A description of all covariates tested
☒	☐	A description of any assumptions or corrections, such as tests of normality and adjustment for multiple comparisons
☐	☒	A full description of the statistical parameters including central tendency (e.g. means) or other basic estimates (e.g. regression coefficient) AND variation (e.g. standard deviation) or associated estimates of uncertainty (e.g. confidence intervals)
☒	☐	For null hypothesis testing, the test statistic (e.g. F, t, r) with confidence intervals, effect sizes, degrees of freedom and P value noted<br>*Give P values as exact values whenever suitable.*
☒	☐	For Bayesian analysis, information on the choice of priors and Markov chain Monte Carlo settings
☒	☐	For hierarchical and complex designs, identification of the appropriate level for tests and full reporting of outcomes
☒	☐	Estimates of effect sizes (e.g. Cohen's d, Pearson's r), indicating how they were calculated

Our web collection on statistics for biologists contains articles on many of the points above.

Software and code

Policy information about availability of computer code

Data collection	BioTek Gen5 microplate reader data collection/analysis software was used to collect the data presented in this manuscript.
Data analysis	GraphPad Prism 8.0.2 was used to analyze all data in the manuscript. DNA constructs design was performed using the NUPACK Tubedesign Algorithm (NUPACK Version 3.2), using the custom analysis code available at: https://github.com/ianapt/PSD_design

For manuscripts utilizing custom algorithms or software that are central to the research but not yet described in published literature, software must be made available to editors and reviewers. We strongly encourage code deposition in a community repository (e.g. GitHub). See the Nature Research guidelines for submitting code & software for further information.

Data

Policy information about availability of data

All manuscripts must include a data availability statement. This statement should provide the following information, where applicable:

- Accession codes, unique identifiers, or web links for publicly available datasets
- A list of figures that have associated raw data
- A description of any restrictions on data availability

The source data underlying Figures 1-4 and Supplementary Figures 2, 4, 6, and 7 are provided as a Source Data File.

Field-specific reporting

Life sciences study design

All studies must disclose on these points even when the disclosure is negative.

Sample size	No sample size calculation was performed. Sample sizes were chosen to be n=3 to ensure that we could detect outliers within the triplicate data to note errors in data acquisition, as well as to allow us to quantify the sample-to-sample variation in our measurements.
Data exclusions	No data was excluded in the analysis of raw or normalized binding curves. In the pH-dependence analysis (e.g. figure 1d) datapoints for pH 4.5 were excluded from curves for the TAT60 and TAT70 constructs. This was done because of substantial uncertainty in the fitted Kd values, which was caused by the high background signal and low binding curve amplitude observed with the dramatically decreased Kd (e.g. supplementary figure 3, TAT60, pH 4.5).
Replication	Replication of the results was attempted once. This attempt at replication produced consistent results. Replication details: pH-dependent ATP binding assays were run for the same constructs on different days or in modified buffer conditions (different salt concentrations and selection of pH-buffering reagnets). These experiments showed similar trends in pH-dependence behavior as observed in the main data with similar pKa and pH-dependence magnitudes as presented in the manuscript, however these assays were either run over the entire pH range tested but with n=1, or over only a subset pH values with n=3.
Randomization	The identity of each sample was known prior to measurement, so no randomization was required.
Blinding	The identity of each sample was known prior to each measurement, so no blinding was required.

Reporting for specific materials, systems and methods

We require information from authors about some types of materials, experimental systems and methods used in many studies. Here, indicate whether each material, system or method listed is relevant to your study. If you are not sure if a list item applies to your research, read the appropriate section before selecting a response.

Materials & experimental systems

n/a	Involved in the study
☒	☐ Antibodies
☒	☐ Eukaryotic cell lines
☒	☐ Palaeontology and archaeology
☒	☐ Animals and other organisms
☒	☐ Human research participants
☒	☐ Clinical data
☒	☐ Dual use research of concern

Methods

n/a	Involved in the study
☒	☐ ChIP-seq
☒	☐ Flow cytometry
☒	☐ MRI-based neuroimaging
